# Digital inclusion and urban-rural integration: Mechanisms and regional heterogeneity in China

**DOI:** 10.1371/journal.pone.0340641

**Published:** 2026-01-20

**Authors:** Ximing Qiu, Ming Yu, Xia Chen

**Affiliations:** 1 College of Public Administration and Law, Hunan Agricultural University, Changsha, China; 2 Hunan Urban Professional College, Changsha, China; Huaqiao University, CHINA

## Abstract

Although digital inclusion is globally recognized as a key pathway to equitable development, rigorous empirical evidence on its specific mechanisms and regionally varying effects in driving urban-rural integration in developing countries remains scarce. To fill this gap, this study employs a fixed-effects model and robustness checks to analyze the impact of digital inclusion on urban-rural integration, using evidence from China. The findings show that digital inclusion enhances urban-rural integration by reducing information asymmetry, improving access to education and healthcare resources, and fostering industrial convergence. However, digital inclusion exerts a significant positive effect on urban-rural integration in eastern and central China, but has no significant effect in western China. These findings challenge the conventional view that digital policies have a homogeneous impact and emphasize the need for spatially differentiated interventions. By integrating an analysis of these mechanisms with an examination of regional disparities, this research advances theoretical understanding of digital inclusion’s societal effects and provides critical empirical support and policy insights for developing countries aiming to develop regionally differentiated digital inclusion strategies.

## Introduction

With the accelerated advancement of global digitalization, digital inclusion has emerged as a central theme in international discourse. It generally refers to providing universally accessible digital technologies, information, and services to ensure all members of society benefit equitably, regardless of their economic, geographical, or social backgrounds. Beyond technological advancement alone, it encompasses critical dimensions of social equity, economic development, and educational opportunities [1]. Globally, numerous countries and regions have integrated it into their social development strategies. For example, since 2010, the European Union (EU) has implemented policies to reduce the digital divide, increase technology penetration, and promote cross-regional digital inclusion via its Digital Single Market strategy. In its 2030 Sustainable Development Goals, the United Nations (UN) explicitly prioritizes using ICT accessibility and digital advancement to narrow the global wealth gap [2] and advance inclusive growth worldwide. Additionally, the Organization for Economic Co-operation and Development (OECD) notes in its reports that advancing digital inclusion is not only essential to boost economic efficiency, but also a key strategy for achieving social equity and promoting an inclusive economy. Thus, as a central theme in modern societal development, it has garnered significant attention from governments and international organizations worldwide and has become a vital tool for integrating diverse social groups.

Empirical evidence from countries worldwide shows that digital inclusion not only facilitates the sharing of information and resources, but also accelerates urban-rural integration and reduces development disparities. For example, Nordic countries like Finland and Sweden have significantly narrowed the urban-rural digital divide by ensuring universal access to high-quality internet services and digital skills training. Their policy interventions include subsidizing internet access in remote areas [3], integrating digital technologies into public services, and strengthening digital literacy training. These measures have collectively improved digital competencies among rural and urban populations, enabled more equitable allocation of public resources, reduced development gaps, and accelerated urban-rural integration. In Asia, the Republic of Korea (ROK)‘s Digital Village Initiative has promoted widespread adoption of information technologies in rural areas [4], providing e-commerce platforms, smart healthcare, and digital education services. These services have significantly improved rural residents’ quality of life and created more employment opportunities. Similarly, India’s Digital India program—by providing affordable smartphones and internet access—has improved nationwide digital connectivity, particularly in rural areas [5]. This program has increased rural residents’ access to education, healthcare, and financial services, while stimulating the flow of social capital and promoting balanced regional development. Collectively, these international experiences show that implementing digital inclusion policies can effectively promote urban-rural integration and offer a critical pathway to addressing regional imbalances.

As the world’s largest developing economy, China faces persistent urban-rural disparities as a critical challenge in its socioeconomic development. With accelerated digital transformation, digital inclusion has gained growing recognition from the government and academia as a vital pathway to foster urban-rural integration and advance equitable development [6]. In recent years, China has implemented comprehensive policy initiatives, including the “Internet Plus” strategy, the “Rural Revitalization” strategy, and the “Digital Rural Development” program, designed to accelerate digitalization and promote urban-rural integration. These policies not only cover infrastructure upgrades but also aim to improve digital literacy and skills among rural residents and promote the wider adoption of digital technology in agriculture, education, healthcare, and other sectors. China’s digital inclusion policy also focuses on promoting the equitable distribution of digital resources and achieving a balance between information sharing and resource flows by increasing support for poorer regions. However, significant challenges persist in promoting digital inclusion, including underdeveloped rural infrastructure, insufficient digital skills training, and persistent digital literacy gaps between urban and rural populations [7].

Therefore, there are several key questions that need to be addressed urgently to integrate digital inclusion into the urban-rural integration agenda. First, although digital technologies have been widely adopted in practice, the specific pathways through which they promote urban-rural integration, such as information flow, educational resource sharing, medical resource allocation, and industrial convergence, remain inadequately explored and systematically articulated. Second, the effects of digital inclusion vary across regions; however, the extent and underlying mechanisms of these spatial disparities remain poorly understood. Third, within the context of China, characterized by its persistent urban-rural dual structure and rapid digital transformation, there is an urgent need to provide targeted, empirical evidence to inform both theoretical debates and policy design. Accordingly, this study aims to develop a multidimensional analytical framework of digital inclusion, empirically examine its mechanisms influencing urban–rural integration, and further explore regional heterogeneity. The findings are expected to provide theoretical insights and inform the design of differentiated digital inclusion strategies.

## Literature review and research hypotheses

### Literature review

#### Conceptual connotation and research evolution of digital inclusion.

As an interdisciplinary field spanning social, technological, and economic dimensions, digital inclusion has garnered increasingly significant global attention from both academics and policymakers. The core philosophy of digital inclusion is to eliminate digital divide-driven social inequalities by expanding access to information technologies. This effort specifically targets marginalized populations and rural communities, with the ultimate goal of advancing inclusive growth and equitable development [8]. The United Nations originally advanced this concept, which is now enshrined in its digital development goals as a key strategy for building inclusive societies. It has evolved from basic technology diffusion to a comprehensive policy construct. This construct encompasses infrastructure accessibility, service affordability, interface usability, and application integration across social service ecosystems. Scholars have interpreted the concept through diverse theoretical lenses, leading to multidimensional frameworks. Some studies [9] suggest that digital inclusion transcends mere technology diffusion and necessitates cultivating digital literacy to ensure meaningful societal participation. Others emphasize the role of institutional frameworks [10], contending that effective implementation requires context-specific policies and targeted support for vulnerable groups to prevent the exacerbation of existing inequalities. Collectively, these perspectives highlight that digital inclusion is not merely a technical undertaking but also a critical socioeconomic governance challenge.

The scholarship on digital inclusion has evolved through distinct conceptual phases. Early research centered on the analysis of the digital divide, emphasizing the asymmetrical distribution of information technologies and its ramifications for social stratification. It focused primarily on the direct impact of digital technologies on socio-economic development and explored technical solutions to bridge access gaps. As research evolved, scholars recognized digital inclusion not merely as a technological challenge but as a complex sociopolitical process that requires multifaceted policy, cultural, and educational approaches. Consequently, contemporary research has shifted toward institutional and structural analyses [11], examining digital inclusion’s intersections with public policy frameworks, social capital formation, and welfare systems. This paradigm has generated theoretical models that conceptualize digital inclusion as a catalyst for equitable development and societal transformation. Accelerating global digitalization in recent years has further expanded scholarly horizons. Current research focuses on the roles of governmental policies, corporate responsibility, and social innovation in implementing digital inclusion. Concurrently, comparative cross-national studies explore context-specific pathways for advancing digital equity and social cohesion. Thus, the research domain has transitioned from a narrow technical focus to a multidimensional governance challenge over the years.

#### Connotation of urban-rural integration and the pathway to its realization.

Urban-rural integration refers to the coordinated development that spans across economic, social, cultural, and resource dimensions. It aims to eliminate spatial disparities by facilitating the unrestricted flow and optimal allocation of resources, industries, populations, and other production factors. A central objective is to transcend historically entrenched urban-rural dual structures [12] and thereby to achieve balanced convergence in economic advancement, social progress, and cultural continuity. Unlike conventional urban-rural unification models, urban-rural integration emphasizes multidimensional strategies, encompassing institutional arrangements, policy design, and social coordination mechanisms, to facilitate synergistic and optimal resource allocation between urban and rural areas. Early theories of urban-rural integration predominantly centered on policy analysis and case studies, focusing on industrial realignment strategies to extend urban development benefits to rural hinterlands. However, accelerated economic globalization and regional integration have increasingly shown that urban-rural integration transcends mere resource allocation [13]. It requires addressing complex socio-institutional structures; consequently, contemporary research must not only focus on economic factor mobility but also identify the optimal balance of regional development across regions and explore sustainable frameworks for enhancing social interdependence.

Achieving urban-rural integration essentially requires a comprehensive strategy that encompasses economic drivers, policy instruments, social welfare, and cultural identity. First, economic drivers are pivotal to this integration, particularly in the context of digitalization [14]. Digital technologies facilitate the optimal allocation of resources and the integration of industrial chains by transforming inter-regional division of labor, resource allocation, and production modes, thereby serving as a critical driver. Second, proactive government policies exert decisive influence on agricultural modernization, infrastructure development, and public service delivery. The government can enhance rural development potential while narrowing urban-rural development gaps by rationalizing land policies, increasing investment in agricultural technology, and implementing regional coordination strategies. Furthermore, the improvement of the social welfare system is another critical pathway for promoting urban-rural integration. A robust social security system can effectively reduce urban-rural income disparities, elevate rural living standards, and provide institutional safeguards for urban-rural integration. Finally, strengthening cultural identity is vital for this integration; preserving and revitalizing rural cultural heritage fosters a shared sense of identity and belonging, which promotes harmonious socio-spatial relations. In conclusion, urban-rural integration is a complex systemic project that requires collective coordinated efforts from government, market, and civil society to successfully align its economic, policy, social, and cultural dimensions.

#### Impact relationship between digital inclusion and urban-rural integration.

Digital inclusion serves as a key mechanism for promoting social equity and urban-rural integration [15]. Its value stems not only from popularizing technology but also from innovatively ensuring equal access for both urban and rural populations to social, economic, and cultural resources. Empirical evidence indicates that digital inclusion can significantly reduce information acquisition costs, improve service accessibility, and narrow the urban-rural development gap by breaking down resource barriers in fields such as education and healthcare [16]. Therefore, it effectively provides an effective pathway to addressing both the information divide and resource inequality. This is particularly critical in the contexts of rural revitalization and social governance, where digital inclusion can stimulate the flow of social capital between urban and rural areas and empower rural communities through enhanced participation and autonomous development. Therefore, digital inclusion is not merely a technical issue but a key driver of innovation in social systems and public policy, thereby playing a vital role in achieving urban-rural integration.

Furthermore, digital inclusion significantly influences urban-rural integration by promoting economic restructuring and industry chain integration. The popularization and application of digital technology have established digital inclusion as a key driver of industrial convergence between urban and rural areas. Digital inclusion provides urban and rural areas with broader market access channels and economic cooperation opportunities [17]. Particularly in rural areas, the rapid growth of e-commerce, telecommuting, and digital agriculture breaks down the resource barriers inherent in the traditional urban-rural economic model and further fosters synergistic industrial development. Digital platforms and internet infrastructure enable more efficient allocation and mobilization of production factors between urban and rural areas, transforming the economic structure toward diversification and innovation. Moreover, by promoting technological innovation, digital inclusion reduces the digital divide, which in turn enhances industrial competitiveness and labor productivity in rural areas [18]. This is evident in agriculture, where agricultural digital transformation improves production efficiency and optimizes resource allocation, thereby promoting the integration of agriculture with other industries and synergistic growth between urban and rural economies. Thus, as a key driver of urban-rural integration, digital inclusion facilitates the equal sharing of social resources and fosters balanced economic development, laying the foundation for a more sustainable urban-rural development model.

#### Literature review.

International research has generated a considerable body of evidence on digital inclusion, urban-rural integration, and the interrelationship between them. This work establishes a robust theoretical foundation and offers valuable practical insights. Research on digital inclusion has provided critical insights for addressing the digital divide and associated social inequalities. Specifically, scholars have examined the mechanisms and pathways through which digital inclusion fosters urban-rural integration, including the technological, policy, and social welfare dimensions. Concurrently, research on urban-rural integration has developed systematic theoretical frameworks for exploring the development models of urban-rural integration, especially in designing pathways for economic, social, and cultural integration, thereby generating innovative approaches. Furthermore, investigations into the relationship between digital inclusion and urban-rural integration have elucidated their intrinsic connections and synergistic mechanisms, thus providing a solid academic foundation for future research in this area.

Overall, existing research on digital inclusion, urban-rural integration, and their interrelationship has yielded substantial findings, thus establishing a theoretical and practical foundation. However, three limitations are evident in the current research. First, most studies focus on developed countries, lacking in-depth analysis of China as a typical large developing nation. Second, methodological approaches often remain confined to theoretical deduction or policy discussions, lacking systematic empirical verification. Third, while the role of digital inclusion in promoting urban-rural integration is acknowledged, its specific mechanisms and regional heterogeneities have not been adequately explained. This study aims to make several contributions to the literature. Theoretically, we intend to develop a framework: “information flow—educational resource sharing—medical resource allocation—industrial convergence” centered on multiple pathways to clarify the underlying mechanisms. Methodologically, a multidimensional digital inclusion index encompassing infrastructure, innovation capacity, and consumption capacity will be constructed. This index will be aligned with an integrated measurement system for urban-rural integration, thereby enhancing the operational feasibility and comparability of the research. Empirically, we address a critical gap by providing systematic evidence from China and testing the mechanisms and their regional variations by using inter-provincial panel data. These contributions collectively broaden the theoretical perspectives and deliver targeted empirical insights for policy-making.

### Research hypotheses

#### Mechanisms of digital inclusion driving urban-rural integration.

Digital inclusion, as a key pathway to promoting urban-rural integration, aligns with the fundamental principles of the Social Capital Theory and the Technology Acceptance Model in social sciences relevant to urban-rural development. According to the Social Capital Theory [19], the building of social networks and social trust is crucial for social integration and resource flow. Digital inclusion bridges the urban-rural divide in information access and technology adoption by expanding access to information technology in rural areas, thereby enhancing rural residents’ sense of participation and belonging within the broader information and resource flows [20]. This technological access not only enhances economic and social participation in rural areas but also promotes the accumulation of social capital, enabling rural regions to better integrate into the broader societal network. Furthermore, the Technology Acceptance Model [21] posits that technology adoption is not merely an individual acceptance process but also a systemic transformation at the societal level. In this process, the widespread adoption of digital technology has enabled rural residents to conveniently access public services such as telemedicine, online education, and digital finance. This has not only improved the quality of life and well-being of rural residents but also narrowed the urban-rural gap in public services such as education, healthcare, and finance [22]. Especially in emerging fields such as e-commerce and digital agriculture, the widespread adoption of such digital technology has driven the transformation and upgrading of the rural economy, creating new employment opportunities and economic growth points, thereby accelerating the further integration of urban-rural economies. The Digital Divide Theory further emphasizes that the widespread adoption of information technology is a key factor in reducing social inequality. Digital inclusion not only provides rural residents with equal access to social services but also promotes urban-rural collaboration across social, economic, and cultural levels by enhancing resource mobility and optimizing cross-regional resource allocation. In summary, digital inclusion serves as a core support system for promoting urban-rural integration. It not only bridges the urban-rural divide but also provides both theoretical and practical foundations for achieving social equity and advancing sustainable development.

Accordingly, Hypothesis 1 (H1) is proposed: Digital inclusion exerts a positive influence on urban-rural integration as a whole.

#### Information flow as the fundamental medium for urban-rural integration.

Digital inclusion promotes urban-rural integration by accelerating the flow of information and enhancing communication and interaction between urban and rural areas. This provides a theoretical foundation for the urban-rural integration process, aligning with the Information Society Theory and the Digital Divide Theory in social sciences. According to the Information Society Theory [23], the flow and acquisition of information serve as the core driving force for the social structure and economic development. As a key tool for the flow of information, digital technology effectively breaks down information barriers between urban and rural areas. Within the context of digital inclusion, the development of information technology enables rural areas to access the internet easily, thereby allowing residents in these areas to obtain public resources—such as education, healthcare, and finance—that were previously more accessible in urban areas. This process fundamentally alleviates the information divide between urban and rural areas. The Digital Divide Theory [24] posits that the differential access to information technology is a root cause of inequality across regions, and among social strata. By narrowing this gap, digital inclusion not only promotes rural residents’ equitable access to public resources such as education and healthcare but also provides a crucial platform for cultural and economic interaction between urban and rural areas. The widespread adoption of the internet enables urban and rural residents, regardless of geographical location, to readily access timely information on policies, markets, society, and other areas, thus breaking the traditional information isolation and reducing the negative impacts of the digital divide. Particularly in the field of education, the widespread application of digital resources enables rural students to access the same high-quality educational resources as their urban counterparts through online platforms. This not only helps narrow the urban-rural gap in areas such as education and healthcare but also promotes economic integration between urban and rural areas [25]. Furthermore, the enhanced flow of information has improved the efficiency of social resources sharing, breaking down geographical barriers to resources and promoting interaction and collaboration between urban and rural areas. Ultimately, digital inclusion provides a more robust foundation for urban-rural integration, thereby contributing to the development of a more balanced social structure across social, economic, and cultural dimensions. Thus, it serves not only as an effective means of narrowing the urban-rural gap but also as a crucial pathway to further promoting urban-rural integration and social equity.

Accordingly, we propose Hypothesis 2 (H2): Digital inclusion promotes urban-rural integration through the mediating role of the enhanced flow of information.

#### Synergistic pathways of educational resource sharing in promoting urban-rural integration.

According to Bourdieu’s theory of cultural capital [26], education serves as a crucial field for social class reproduction, and the access to and utilization of educational resources directly influences an individual’s social mobility and development opportunities. Digital inclusion bridges the urban-rural gap in education by enhancing access to information technology in rural areas. This enables students and teachers in rural regions, through internet platforms, to access high-quality educational resources equivalent to those available to urban students. With the increase in rural broadband access rates [27], the widespread availability of digital educational content has become achievable. Students in rural areas are no longer confined to traditional educational models, but can access broader learning opportunities through online platforms, prerecorded digital courses, and distance education. This sharing of resources has not only improved the academic performance of rural students but also driven an overall improvement in educational quality. The application of digital technology has facilitated the sharing and flow of educational information, breaking down geographical barriers that restrict access to educational resources between urban and rural areas. It has reduced the prevalent concentration of resources in traditional education systems, thereby achieving a more equitable distribution of educational resources. Digital inclusion has contributed to a marked improvement in educational attainment in rural areas. This progress has not only enhanced the cultural capital in these regions but also fostered mutual advancement in urban and rural education, which in turn has accelerated urban-rural integration. Thus, digital inclusion not only provides rural areas with pathways to access cultural capital but also offers robust support for achieving educational equity and social integration.

Accordingly, we propose Hypothesis 3 (H3): Digital inclusion promotes urban-rural integration by further enhancing the sharing of educational resources.

#### Balanced dimensions of medical resource allocation to optimize urban-rural integration.

According to the Social Integration Theory [27], the participation of social members and the equitable distribution of resources are crucial for promoting social stability and progress. In the field of healthcare, digital inclusion provides urban and rural residents with more equitable access to medical services. The widespread adoption of internet-based telemedicine services [28] has enabled rural residents to access high-quality healthcare resources more conveniently. With the sustained increase in broadband internet access rates and the digital transformation of relevant healthcare services, medical resources have been effectively circulated between urban and rural areas. Through telemedicine services, residents in rural areas can directly obtain diagnoses from urban specialists via the internet platforms [29], which addresses the challenges of limited access to affordable healthcare in these regions and effectively bridges the urban-rural gap in medical services. Moreover, the application of digital technologies has facilitated both the sharing of medical information and the efficient utilization of healthcare resources. In resource-scarce rural areas, digital technologies can help optimize the allocation and utilization of healthcare resources, thereby promoting a more balanced geographical distribution of healthcare services. By enhancing urban-rural interaction and resource flow, digital inclusion enhances the quality of healthcare in rural areas, promotes a more equitable distribution of medical services, and advances the social benefits linked to urban-rural integration. Therefore, digital inclusion provides robust support for achieving urban-rural integration, highlighting the importance of equitable healthcare resource allocation to social cohesion.

Accordingly, we propose Hypothesis 4 (H4): Digital inclusion promotes urban-rural integration by further optimizing the allocation of healthcare resources.

#### Industrial convergence restructuring urban-rural integration as a structural driver.

Structural Functionalism [30] posits that components of society are interdependent and operate collectively to ensure the stability and development of the overall social system. Within this theoretical framework, the widespread adoption of digital technologies has enabled the integration of industrial and value chains between urban and rural areas, particularly in sectors such as e-commerce and digital agriculture. These technologies have generated new development opportunities for both urban and rural economies [31]. Specifically, as industrial enterprises increase their R&D expenditures, digital technologies and digital innovation have progressively driven the integration of urban and rural industries. Especially with the support of e-commerce platforms, products from rural areas have entered urban markets more easily, while urban capital and technology have flowed into rural areas, thereby driving the development of the rural economy. Meanwhile, the rise of digital agriculture has brought about profound changes in agricultural production methods in rural areas. Digital technologies have enhanced agricultural productivity, optimized resource allocation, and facilitated the transformation and upgrading of rural economies. Digital inclusion has enhanced urban-rural industrial collaboration. Urban-rural economic interaction has provided more opportunities for economic growth, has optimized urban-rural economic structures, and has promoted urban-rural economic integration. This process embodies the synergy and complementarity of urban-rural economic structures, and in turn promotes the overall optimization of the socioeconomic system. Therefore, digital inclusion is not only an instrument for enhancing rural economic development but also a key pathway for promoting urban-rural integration. It highlights the synergistic interactions among various segments of the social system within Structural Functionalism.

Accordingly, we propose Hypothesis 5 (H5): Digital inclusion promotes urban-rural integration by facilitating industrial convergence.

**Fig 1:**
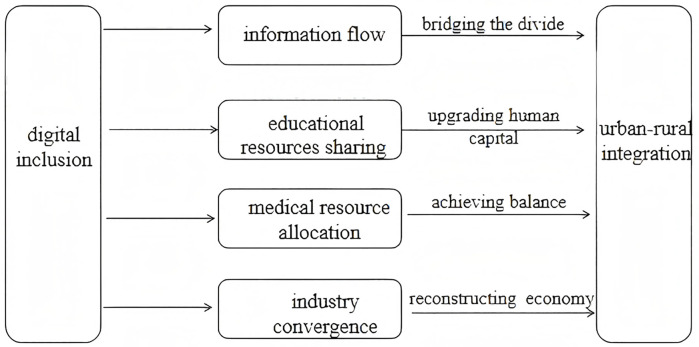
Impact of digital inclusion on urban-rural integration.

In summary, the relationship between digital inclusion and urban-rural integration is illustrated in Fig 1.

## Data sources and variable selection

### Data sources

The data sources for this study are as follows: First, the official websites of the National Bureau of Statistics (NBS) offer national-level statistical data, including comprehensive data on population, economy, and society, which supplies the foundation for macro-level analysis. Second, the official websites of provincial statistical bureaus provide detailed statistical data for each province, covering economic, social, and infrastructure-related areas, which forms a critical basis for studying inter-regional differences. Third, County Statistical Yearbooks deliver detailed data for in-depth analysis of socioeconomic conditions, infrastructure development, and public services at the county and district levels, which reflects micro-differences in urban-rural integration. Fourth, the Education Statistical Yearbook furnishes detailed national and regional data on educational resource distribution, educational quality, and educational investment, which is valuable for studying digital inclusion’s role in educational resource sharing. Fifth, Industrial Statistical Yearbooks, especially the China Industrial Statistical Yearbook, offer necessary data for analyzing digital inclusion’s impact in the industrial sector, particularly regarding enterprises’ R&D investment and technological innovation. Finally, the China National Statistical Yearbook, a comprehensive and authoritative statistical source, presents annual economic, social, and developmental data for the nation, provinces, and municipalities, which serves as a key data source for this study. This integrated dataset enables a comprehensive assessment of digital inclusion’s impact on urban-rural integration and provides empirical evidence for policy optimization.

This study focuses on the period from 2006 to 2019, a period characterized by significant policy and practical transformations in China regarding digital inclusion and urban-rural integration. We excluded data prior to 2006 because digital technology adoption remained limited in rural China, with relevant policies and infrastructure remaining underdeveloped. Consequently, data from this period have limited applicability in analyzing digital inclusion’s impact on urban-rural integration. Beginning in 2006, national initiatives such as the Broadband China strategy and the Digital Rural Development program accelerated the development of digital infrastructure across the country. This infrastructure expansion promoted the penetration of information technologies and digital services into rural areas, thereby gradually altering the urban-rural digital divide. We selected 2019 as the endpoint for two main reasons. First, by 2019, most relevant policies had been implemented and produced measurable outcomes, providing robust data for analysis. Second, post-2019 developments represent an ongoing phase of digital technology maturation and policy refinement, whose impacts require further longitudinal assessment. Thus, selecting the period from 2006 to 2019 ensures the integrity of data and the interpretability of research findings. It also enables us to capture the direct impact of digital inclusion policy implementation on urban-rural integration more clearly.

### Dependent variable: Urban-rural integration index

We measured the urban-rural integration level by constructing a comprehensive evaluation system, based on the frameworks and indicators from prior literature [24–27]. As shown in Table 1, this system encompasses four key dimensions: spatial integration, economic integration, social integration, and ecological integration. Specifically, we selected 13 indicators, including urban-rural population density, income gap, Engel’s coefficient difference, highway connectivity mileage, disparities in educational and medical resource distribution and so on. These indicators comprehensively reflect the level of urban-rural integrated development, thereby providing a solid foundation for subsequent empirical analysis.

**Table 1:** Indicators of urban-rural integration level.

Primary Indicator	Secondary Indicator	Tertiary Indicator	Attribute	Max	Min
Urban-Rural Integration	Spatial Integration [32]	Urban Resident Population (10,000 persons)	+	9466	58
		Total Resident Population (10,000 persons)	+	12684	280
		Urban Built-up Area (km²)	+	6583	75
		Highway Network Length (10,000 km)	+	39.89	0.81
	Economic Integration [33]	Per Capita Disposable Income of Urban Households (yuan)	+	82429	8013
		Per Capita Disposable Income of Rural Households (yuan)	+	38521	1971
		Local Government Social Security & Employment Expenditure (yuan)	+	51295	5960
		Local Government General Budget Expenditure (yuan)	+	27205	1658
	Social Integration [34]	Per Capita Consumption Expenditure of Urban Residents (yuan)	+	53700	0.53
		Per Capita Consumption Expenditure of Rural Residents (yuan)	+	33026	615
		Number of Beds in County-Level Social Welfare Institutions (units)	+	2131.89	90.57
		Number of Compulsory Education Schools	+	18247.01	151.24
	Ecological Integration [35]	Forest Area (1,000 hectares)	+	2614.85	5.97
		Daily Wastewater Treatment Capacity (10,000 tons)	+	2929.7	5

Note:

1. The “Attribute” column indicates the hypothesized direction of influence on the overall integration index, where “+” denotes a positive contribution.

2. [28–31] refer to the indicator definition from existing literature, see References for details.

### Core explanatory variable, control variable and mediating variable

#### Core explanatory variable: Digital inclusion.

Digital Inclusion (DI): This study draws on existing theoretical frameworks, empirical research, and the measurement indicators and research methods proposed by scholars [32, 36–38]. Informed by the core dimensions of digital inclusion, our comprehensive evaluation system encompasses three key areas: infrastructure inclusion, innovation capacity inclusion, and consumption capacity inclusion [39–42]. Specifically, this system comprises core indicators grouped by the three sub-dimensions: (1) Infrastructure Inclusion: total Internet broadband subscribers, urban broadband subscribers, and rural broadband subscribers (e.g., per 100 inhabitants); (2) Innovation Capacity Inclusion: R&D expenditures of industrial enterprises above designated size and number of valid invention patents held by these enterprises; (3) Consumption Capacity Inclusion: urban and rural residents’ online consumer price indices. All indicators are detailed in Table 2.

**Table 2:** Core Explanatory Variable Indicators.

Primary Indicator	Secondary Indicator	Tertiary Indicator	Attribute	Max	Min
Digital Inclusion	Infrastructure Inclusion [43]	Broadband Internet Subscribers (10,000 households)	+	4277.7	4.9
		Urban Broadband Subscribers (10,000 households)	+	3243.7	5.8
		Rural Broadband Subscribers (10,000 households)	+	1560.7	0
	Innovation Capacity Inclusion [44]	R&D expenditure of industrial enterprises above designated size (10,000 yuan)	+	29021849	1637
		Valid Invention Patents of Above-designated-size Manufacturing Firms (units)	+	511717	19
	Consumption Capacity Inclusion [45]	Urban E-commerce Consumer Price Index (Standardized)	+	4.369	−2.536
		Rural E-commerce Consumer Price Index (Standardized)	+	1.192	−0.976

Note: The “Attribute” column indicates the hypothesized direction of influence on the overall integration index, where “+” denotes a positive contribution.

#### Control and mediating variables.

We examined four mediating variables and four control variables to elucidate the process by which digital inclusion promotes urban-rural integration. First, the number of beds in medical and health facilities per 10,000 people in each province (MedShare_it) serves as a mediating variable, directly reflecting disparities in health resources between urban and rural areas. The widespread adoption of digital technology and telemedicine [46] enhances the mobility and accessibility of healthcare services across urban and rural areas. In particular, information technology-enabled support enables rural areas to access medical services from urban specialists more easily, alleviating the shortages of medical resources in rural areas. We used the per capita urban road area (unit: square meters; URP) as a control variable to control for differences in transportation infrastructure connectivity between urban and rural areas. Improved transportation facilitates the efficient flow of resources and promotes a more balanced distribution of healthcare services across urban and rural areas [47]. The second mediating variable, the ratio of rural per capita consumption expenditure on transportation and communication to rural per capita disposable income (InfoFlow_it), reveals the potential and obstacles to information flow and reflects levels of investment in information and communication facilities in rural areas. The advancement of digital inclusion has continuously expanded information channels in rural areas, narrowing the urban-rural information gap. The control variable, the total number of employed individuals in rural areas, reveals the vitality of the rural labor market and residents’ capacity to participate in the digital economy [48], further driving the interaction and integration between urban and rural economies. Education funding intensity (e.g., as a share of regional GDP) serves as a mediating variable by facilitating educational resource sharing within digital inclusion frameworks. This process enables rural students to access quality educational resources through online learning platforms comparable to those available in urban areas. We included per capita expenditure on postal and telecommunications service as a control variable, as it represents the foundational infrastructure for information circulation and educational resource dissemination, enhancing the efficiency of resource sharing. Finally, the mediating variable, the ratio of new product development expenditure to GDP (unit: 10,000 yuan), captures innovation dynamics that drive urban-rural industrial convergence [49]. Digital inclusion accelerates the digital transformation of rural economies, particularly in the agricultural and e-commerce sectors. Converging industrial chains resulting from this digital transformation foster synergistic economic development between urban and rural regions. Fiscal expenditure bias (e.g., the ratio of rural fiscal expenditure to total fiscal expenditure), as a control variable variable, influences the policy orientation of government resource allocation. Local fiscal support facilitates the modernization of economic transformation and industrial upgrading in rural regions [50]. Collectively, these mediating and control variables operate through coordinated mechanisms across economic, social, medical, and educational fields to facilitate urban-rural integration. The hypothesized relationships are detailed in Table 3.

**Table 3:** Control and Mediating Variables.

Variable Type	Variable Description	Variable Meaning	Attribute	Max	Min
Mediating Variables	the number of beds in medical and health facilities per 10,000 people in each province (MedShare_it)	Reflects healthcare resource allocation, especially urban-rural distribution disparities to support inclusive health services	+	72.13	0.5
	Ratio of rural household per capita expenditure on transport & ICT (yuan) to rural per capita disposable income (InfoFlow_it)	Measures investment in information flow and transportation infrastructure, indicating accessibility of urban-rural information exchange	+	0.195	0.057
	Ratio of regional public education expenditure to regional GDP (EduShare_it)	Reflects educational investment intensity, promoting urban-rural educational resource equity under digital inclusion	+	0.222	0.099
	Ratio of industrial new product development expenditure to regional GDP (IndShare_it)	Measures industrial innovation vitality and its role in promoting urban-rural industrial convergence	+	0.040	0.002
Control Variables	Urban per capita road area (m²) (URP)	Indicates urban infrastructure development, supporting urban-rural transportation connectivity	+	28	4.04
	Total rural employed population (10⁴ persons) (RE)	Reflects rural labor market dynamism and residents’ capacity to participate in the digital economy	+	1204	1.5
	Total postal & telecom services revenue per province (10⁸ yuan)	Measures information flow penetration, supporting urban-rural information circulation and resource dissemination	+	5807.81	0.24
	Fiscal expenditure bias (ratio of rural fiscal expenditure to total fiscal expenditure, 10⁴ yuan) (FE)	Indicates government fiscal allocation preference toward rural areas, facilitating rural economic transformation	+	18427.01	241.85

Note:

1. The “Attribute” column indicates the hypothesized direction of influence on the overall integration index, where “+” denotes a positive contribution.

2. “_it” denotes provincial-year panel data (i.e., cross-sectional data across Chinese provincial-level administrative regions over multiple years).

3. ICT = Information and Communications Technology; All monetary units are in Chinese yuan (CNY).

## Empirical model construction

### Baseline regression model (Hypothesis 1)

To test Hypothesis 1 that digital inclusion promotes urban-rural integration, we constructed a baseline regression model with Digital Inclusion (DI) as the explanatory variable and the Urban-Rural Integration Level (URI) as the dependent variable. The baseline model is specified as follows:


$$URI_{it}=\alpha +\beta_{\text{1}}DI_{it}+\beta_{\text{2}}CV_{it}+\gamma_{t}+\delta_{i}+\varepsilon \_it\;$$

In this model, *URI*_*it* denotes the urban-rural integration level for province *i* in year *t*, and *DI*_*it* represents the core explanatory variable (i.e., digital inclusion). The term *CV*_*it* represents a vector of control variables, including social, economic, and infrastructure factors that may affect urban-rural integration. We include province-fixed effects (*δ*_*i*) and time-fixed effects (*γ*_*t*) to account for unobserved regional and temporal heterogeneity, and *ε*_*it* is the idiosyncratic error term. This model is used to estimate the association between digital inclusion and urban-rural integration, while it controls various factors such as social, economic, and infrastructure elements that may impact urban-rural integration. The inclusion of time and province fixed effects accounts for unobserved heterogeneity across provinces and over time, ensuring the robustness of our estimates.

### Mediation effect model (Hypotheses 2–5)

To examine the mediating role of information flow (InfoFlow_it) in the relationship between digital inclusion and urban-rural integration, the following regression model is estimated:


$$URI_{it}=\alpha +\beta_{\text{1}}DI_{it}+\beta_{\text{2}}MV+\beta_{\text{3}}CV_{it}+\gamma_{t}+\delta_{i}+\varepsilon \_it$$

In this model, *MV*_*it* represents the mediating variable.

## Empirical testing

### Baseline regression

Table 4 presents the baseline regression results. In the baseline regression analysis, results of regressions with and without control variables reveal the impact of variable inclusion and selection on the magnitude and statistical significance of regression coefficients. The observed changes in regression coefficients indicate that the introduction of control variables significantly improved the model fit and the significance of the regression coefficients. Specifically, the coefficients of some independent variables became significant, and their standard errors decreased, indicating that the control variables effectively reduced the model’s endogeneity and multicollinearity. For example, after controlling for variables, the regression coefficient for URI changed from 0.000 (p = 0.756) to 0.000* (0.000), indicating a higher level of significance. This suggests that after controlling for relevant factors, the influence of URI on the dependent variable becomes more pronounced. Additionally, the coefficients of certain independent variables exhibit significant variation, indicating that the control variables exert multifaceted effects on the regression model. This capability allows for the exclusion of potential confounding factors, thereby enhancing the explanatory power and accuracy of the regression results.

**Table 4:** Baseline Regression and Robustness Tests.

Variables	Baseline Regression		Robustness Tests		
	Without Controls	With Controls	Excluding Municipalities	Random Variable Deletion	Random Period Division
Internet Broadband Subscribers (IBS)	0.03 (0.637)	0.000 (0.756)	0.000 (0.419)	0.000*** (0.000)	−0.008 (0.427)
Urban Broadband Subscribers (UBS)	0.000*** (0.003)	0.000*** (0.000)	0.000*** (0.001)		0.008 (0.415)
Rural Broadband Subscribers (RBS)	−0.05 (0.369)	−0.000 (0.158)	−0.000** (0.045)	−0.000*** (0.000)	0.008 (0.431)
Industrial R&D Expenditure (IRDE)	0.000*** (0.003)	0.000* (0.094)	0.000 (0.102)	−0.000 (0.111)	0.000 (0.116)
Valid Invention Patents of Industrial Enterprises above Designated Size (VIP)	−0.000*** (0.05)	−0.000*** (0.000)	−0.000*** (0.000)		−0.000** (0.033)
Urban Online Consumer Price Index (UO-CPI)	0.014*** (0.000)	0.015*** (0.000)	0.008* (0.092)	0.006 (0.179)	−0.010 (0.272)
Rural Online Consumer Price Index (RO-CPI)	−0.015*** (0.000)	−0.015*** (0.000)	−0.009** (0.025)	−0.008** (0.049)	0.006 (0.373)
Constant	0.286* (0.051)	0.233 (0.121)	0.323** (0.039)	0.393** (0.016)	0.574 (0.257)
Individual/Time Fixed Effects	Yes	Yes	Yes	Yes	Yes
*R2*	0.818	0.830	0.852	0.839	0.816
N	488	372	377	372	372

*Notes: ***, **, * denote significance at 1%, 5%, and 10% levels respectively; standard errors in parentheses. Same applies below.

Regarding significance, p-values in the model with controls are predominantly below 0.05, further indicating that including control variables enhances the significance of the estimated relationships. Compared to the model without controls, the changes in coefficients and standard errors enhance both the credibility of the results and the robustness of the model. Furthermore, the R² values reflect the contribution of control variables to model fit. R² increases from 0.818 in the model without controls to 0.830 in the model with controls. Although modest, this increase is meaningful, demonstrating the control variables’ contribution to enhancing the model’s explanatory power. This indicates that the control variables enable the model to better account for variation in the dependent variable, thus improving overall model fit.

In summary, the baseline regression results indicate that the inclusion of control variables significantly enhances the robustness of the model and the explanatory power of the results. By controlling for external influences, the model specification more accurately captures the relationship between the independent and dependent variables, mitigates omitted variable bias, and thus supports the reliability of the analytical results. These findings provide initial evidence supporting a positive relationship between the independent and dependent variables, thereby confirming Hypothesis 1.

**Fig 2:**
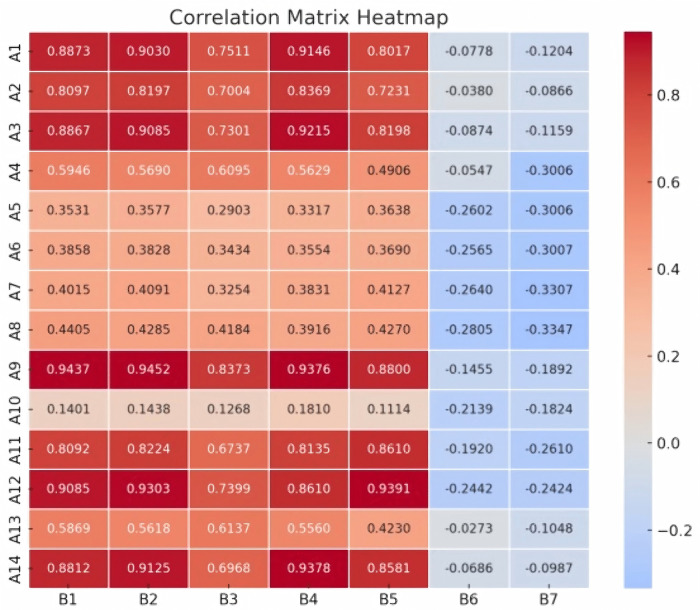
Heatmap of Indicators for Digital Inclusion and Urban-Rural Integration.

To further analyze correlations between indicators of Digital Inclusion (DI) and Urban-Rural Integration (URI), we calculated pairwise correlation coefficients and generated the heat map in Fig 2.

Infrastructure development, especially road mileage (A9) and Internet broadband subscribers (B1), plays a pivotal role in the process of urban-rural integration. Road mileage (A9) reflects the development of the urban-rural transportation network, directly affecting the movement of people and goods and indirectly facilitating information flow by enhancing cross-regional connectivity. Increased road mileage (A9), especially improved rural roads, which are critical for enhancing remote connectivity [51], enables residents in outlying areas to access urban areas more conveniently, thereby promoting urban-rural interaction and communication. Internet broadband subscribers (B1) reflect the popularization of information infrastructure, determining whether urban and rural residents have equal access to digital-era services such as online education, telemedicine, and e-commerce. Improved road infrastructure facilitates rural access to urban internet services by supporting the deployment of broadband infrastructure in rural areas, in turn facilitating information flow, resource sharing, and deepening urban-rural integration. Consequently, road mileage (A9) and Internet broadband subscribers (B1) exhibit a mutually reinforcing relationship. Their synergy in enhancing cross-regional connectivity and information equity provides a robust foundation for urban-rural integration. Furthermore, local general budget expenditures (A12) are crucial to urban-rural integration. Increased local spending on infrastructure, social security, and public services [52] improves the sharing of public resources between urban and rural areas. Particularly in digital infrastructure development, local governments have increased fiscal spending to boost the number of urban broadband subscribers (B2), thereby enhancing the ability of urban residents to access digital public services. As urban broadband becomes more common, the information gap between urban and rural areas narrows. Consequently, rural residents can use the internet to access the same digital resources as urban residents, which promote the balanced social development between urban and rural areas. Therefore, local government expenditures is positively correlated with the number of urban broadband subscribers and is essential for developing digital infrastructure.

In summary, the relationship between road mileage (A9) and Internet broadband subscribers (B1), along with the interaction between local general budget expenditure (A12) and urban broadband subscribers (B2), demonstrates that infrastructure and fiscal policy are central to promoting urban-rural integration. Digital inclusion offers crucial technical support for integration, a process that depends on the material foundation laid by physical infrastructure and enabled by fiscal policy. By improving infrastructure and increasing fiscal investment, integrated urban-rural development can achieve more efficient and balanced social progress.

### Robustness checks

We assessed the robustness of our results using two approaches: first, by estimating our regression models with clustered robust standard errors [53], and second, by conducting robustness tests through random time-period partitioning. We then compared the regression coefficients and corresponding standard errors from both approaches. Overall, this comparison demonstrates that our findings are robust, which supports the reliability of our statistical conclusions.

We employed clustered robust standard errors in our regression analysis to account for intra-cluster correlation in the panel data, which produces more reliable inference by mitigating the impact of autocorrelation within clusters. The analysis identified several statistically significant predictors. Rural per capita consumption showed a positive association with urban-rural integration (coefficient = 0.2153, p = 0.039). Similarly, urban household per capita consumption expenditure was also a significant predictor (coefficient = 0.0410, p = 0.008). Other variables, including urban household per capita disposable income and local government social security and employment expenditure, were also statistically significant. In contrast, the coefficient for urban household per capita disposable income was not statistically significant (p = 0.078), though it approached the conventional threshold. Overall, the use of clustered standard errors confirms that our findings are robust to complex data structures, strengthening the reliability of the analytical results.

The robustness tests based on random time-period partitioning produced results consistent with our main model. Under this method [54], the regression coefficients for most variables remained stable in magnitude, and their standard errors remained small (ranging from 0.000 to 0.001), indicating that the results are largely insensitive to the random partitioning of the time period. Notably, the regression coefficient (−0.0001) for specific variables remained statistically significant under this test, further demonstrating the model’s robustness.

Overall, two distinct robustness tests, clustered robust standard errors and random time-period partitioning, yielded consistent regression results. The stability of the regression coefficients and their standard errors across these tests indicates that the estimated relationships are robust. Consequently, these findings provide confidence in the robustness of our statistical analysis, forming a reliable basis for policy discussion and future research. This robustness strengthens the evidence for our central finding that digital inclusion promotes urban-rural integration, thereby supporting Hypothesis 1.

### Endogeneity tests

We tested for endogeneity using the Durbin-Wu-Hausman test, which compares the coefficient estimates from ordinary least squares (OLS) and instrumental variable (IV) regressions. The test result was not statistically significant (p = 0.158), meaning that we failed to reject the null hypothesis of exogeneity [55] and the endogeneity issues were effectively addressed. Consequently, the OLS estimates are consistent and preferred for interpretation, as we find no evidence of endogeneity bias. Thus, we treat all independent variables as exogenous. The regression results are considered robust against endogeneity concerns.

### Mechanism test and mediation analysis

To test the hypothesized mediating mechanisms (H2-H5) between digital inclusion and urban-rural integration, we performed a mediation analysis following the statistical approach detailed in Section 4.2. The results summarized in Table 5 provide support for these mediating pathways, as indicated by the significant indirect effects.

**Table 5:** Mediation Effects and Transmission Mechanisms.

Variable	Mediation Variable			
	MedShare_it	InfoFlow_it	EduShare_it	IndShare_it
Internet Broadband Subscribers (IBS)	−0.000 (0.979)	0.000 (0.690)	0.000 (0.780)	0.000 (0.747)
Urban Broadband Subscribers (UBS)	0.000*** (0.000)	0.000*** (0.001)	0.000*** (0.000)	0.000*** (0.000)
Rural Broadband Subscribers (RBS)	−0.000 (0.529)	−0.000 (0.145)	−0.000 (0.151)	−0.000 (0.157)
Industrial R&D Expenditure (IRDE)	0.000* (0.052)	0.000* (0.091)	0.000 (0.131)	0.000 (0.104)
Valid Invention Patents of Industrial Enterprises above Designated Size (VIP)	−0.000*** (0.001)	−0.000*** (0.000)	−0.000*** (0.000)	−0.000*** (0.000)
Urban Online Consumer Price Index (UO-CPI)	0.016*** (0.000)	0.015*** (0.000)	0.015*** (0.000)	0.015*** (0.000)
Rural Online Consumer Price Index (RO-CPI)	−0.017*** (0.000)	−0.015*** (0.000)	−0.015*** (0.000)	−0.015*** (0.000)
MedShare_it	0.380** (0.013)			
InfoFlow_it		0.103 (0.334)		
EduShare_it			0.007** (0.033)	
IndShare_it				−0.019 (0.875)
Constant	0.254* (0.083)	0.222 (0.140)	0.177 (0.242)	0.234 (0.120)
Individual/Time Fixed Effects	Yes	Yes	Yes	Yes
*R2*	0.825	0.832	0.833	0.831
N	488	488	488	488

The results for each mediator (MedShare_it, InfoFlow_it, EduShare_it, IndShare_it) are consistent with their respective hypotheses, thus supporting all four (H2-H5). For the mediator InfoFlow_it, urban broadband subscribers (UBS) has a significant positive coefficient (coefficient = 0.000 *, p = 0.001). The effects of other independent variables are also aligned with expectations. These findings suggest that digital inclusion, facilitated by expanded internet access and urban broadband infrastructure, enhances information flow and urban-rural exchange, which in turn promotes urban-rural integration [56], thereby supporting Hypothesis 2. Second, digital inclusion enhances urban-rural integration by facilitating educational resource sharing. For the mediator EduShare_it, the Urban Online Consumer Price Index (UOCPI) shows a positive and statistically significant association (coefficient = 0.015 **, p < 0.01). In contrast, the coefficient for industrial R&D expenditure (IRDE) is not statistically significant at the conventional 0.05 significance level (0.000, p = 0.052). This indicates that digital inclusion promotes educational resource sharing between urban and rural areas, and thus enhances educational equity, which supports Hypothesis 3. Furthermore, digital inclusion promotes urban-rural integration through the optimization of medical resource allocation. For the mediator MedShare_it, Valid Invention Patents (IVP) of industrial enterprises above designated size show a significant negative association (coefficient = −0.000 ***, p = 0.001). Although this finding contradicts initial expectations, it indicates that digital inclusion still exerts a significant impact on the allocation of medical resources. Specifically, enhanced information flow and medical resource sharing driven by digital inclusion contribute to a more balanced distribution of medical resources between urban and rural areas [57]. Therefore, these findings support Hypothesis 4. Finally, digital inclusion promotes urban-rural integration by driving industrial convergence. For the mediator IndShare_it, a positive correlation was observed between R&D expenditures of industrial enterprises above designated size and industrial sharing (IndShare_it) (coefficient = 0.0004, *p* = 0.052), suggesting that digital technology-enabled R&D innovation drives urban-rural industrial convergence. Specifically, it indicates that increased industrial R&D investment may facilitate supply chain integration and resource sharing, thereby advancing urban-rural economic convergence. Accordingly, these results support Hypothesis 5.

In summary, all four hypotheses (2–5) receive empirical support. Digital inclusion promotes interaction and coordinated urban-rural development by accelerating information flow, enhancing educational resource sharing, optimizing medical resource allocation, and facilitating industrial convergence. The statistically significant mediation effects collectively demonstrate that digital inclusion robustly supports urban-rural integration, advancing the convergence process.

### Heterogeneity analysis

#### Regional heterogeneity analysis.

Table 6 displays the results of regional heterogeneity analysis examining the impact of digital inclusion (DI) on urban-rural integration (URI) in eastern, central, and western China. Results indicate a statistically significant positive relationship between DI and URI in both eastern and central regions, but no significant effect in the western region [58]. In the eastern region, the DI coefficient is 0.026 (SE = 0.003, p < 0.001), indicating that DI exerts a significantly positive effect on URI. Similarly, the central region has a DI coefficient of 0.034 (SE = 0.001), also confirming a significant positive relationship between its urban-rural integration index and the dependent variable. Conversely, the western region has a DI coefficient of 0.005 (SE = 0.368, p > 0.05), suggesting that DI has a negligible and statistically insignificant impact on URI [59]. This discrepancy likely reflects regional variations in the mechanisms and intensity of DI’s impact on urban-rural integration. The eastern and central regions may benefit from stronger policy support and higher economic development levels, which in turn amplifies the positive effect of DI on URI.

**Table 6:** Regional heterogeneity analysis.

Variable	Eastern Region	Central Region	Western Region
Urban-Rural Integration *(URI)*	0.026*** (0.003)	0.034*** (0.001)	0.005 (0.368)
Constant	−0.266 (0.422)	−0.276 (0.461)	0.371* (0.092)
Controls	Yes	Yes	Yes
*R2*	0.945	0.903	0.881

Furthermore, the constant term is negative in the eastern (−0.266) and central (−0.276) regions but positive in the western region (0.371), which indicates that the western region has a relatively solid foundation for urban-rural integration. It is possible that specific policies or socioeconomic contexts in the western region have narrowed the urban-rural gap. Here, a positive constant term indicates a stronger facilitating effect of infrastructure and the social environment on the dependent variable (URI) [60]. Conversely, negative constant terms in the eastern and central regions may reflect higher economic development levels coupled with persistent urban-rural disparities. Despite better urban-rural integration performance in these regions, underlying structural barriers still persist.

The R² values for the eastern, central, and western regions were 0.945, 0.903, and 0.881, respectively, indicating the highest explanatory power of the model in the eastern region. The lower goodness-of-fit in the central region and western region suggests weaker explanatory power, potentially due to greater data complexity, regional economic heterogeneity, and variations in local policies. Overall, the effect of digital inclusion (DI) on urban-rural integration (URI) is most pronounced in the eastern region, while its impact is relatively weaker in the western region, reflecting the diverse effects of DI on URI across different regional contexts.

#### Digital literacy heterogeneity analysis.

In this study, we classify regions in mainland China into high- and low-digital-literacy regions based on their digital literacy levels. High-digital-literacy regions consisted primarily of the economically developed eastern provinces, with the addition of Guizhou Province due to its high digital literacy score. These regions exhibit high levels of digital technology adoption, and residents have strong capabilities in accessing, utilizing, and processing digital technologies, enabling them to better adapt to the demands of digital transformation. Low-digital-literacy regions include the central and western regions of mainland China. Digital infrastructure in these regions lags behind that in high-digital-literacy regions, and residents have relatively low digital literacy. The widespread adoption of digital technology faces greater challenges. Within this framework, the impact of digital inclusion on urban-rural integration differs significantly between high- and low-digital-literacy regions (Table 7).

**Table 7:** Digital literacy heterogeneity analysis.

Variable	High-digital-literacy regions	low-digital-literacy regions
URI	0.013*** (0.003)	0.006** (0.002)
Constant	−0.735*** (0.153)	−0.456 (0.462)
Controls	Yes	Yes
R2	0.995	0.919

Our data show that digital inclusion (DI) exerts a statistically significant and strong effect on the dependent variable in high-digital-literacy regions, with a regression coefficient of 0.013 (p < 0.01). This indicates that increased digital inclusion significantly promotes the dependent variable in these regions [61]. For low-digital-literacy regions, the regression coefficient is 0.006. While statistically significant (p < 0.05), this effect is relatively weak, indicating that digital inclusion policies have limited effectiveness in these regions [62–65]. Furthermore, the constant term for high-digital-literacy regions is −0.735 and statistically significant, indicating a low baseline level of the dependent variable when control variables are held at zero. In contrast, the constant term for low-digital-literacy regions is −0.456 and not statistically significant, suggesting a higher but non-significant baseline level of the dependent variable [66].

## Research findings and contributions

### Main findings

First, we identify several key mechanisms—including information flow, educational resource sharing, healthcare resource allocation, and industrial convergence—through which digital inclusion facilitates urban-rural integration. This multifaceted approach contrasts with previous studies that relied on single-dimensional analyses. Furthermore, our findings challenge the prevailing perspective that equates digital inclusion solely with infrastructure development. Our research demonstrates that digital inclusion not only narrows the urban-rural divide through enhanced technological access but also drives coordinated regional development by providing equitable digital service opportunities and promoting the balanced distribution of educational and medical resources. Diverging from studies that merely conceptualize digital inclusion as technological proliferation, our study underscores its role in advancing social equity and optimizing resource allocation. Additionally, digital inclusion promotes urban-rural synergistic growth and narrows the urban-rural economic gap by facilitating the digital transformation of rural industries, particularly digital agriculture and rural e-commerce. This multifaceted mechanism—under-explored in existing research—enriches the theoretical framework linking digital inclusion and urban-rural integration.

Second, we identified the pathways through which digital inclusion facilitates urban-rural integration. Our analysis demonstrates that digital inclusion influences urban-rural integration through multiple mediating variables, beyond direct economic effects. Diverging from conventional linear causality models, our analysis delineates how digital inclusion indirectly promotes urban-rural integration by facilitating information flow, enabling educational resource sharing, and optimizing healthcare resource allocation—mechanisms we identified through rigorous mediation analysis. First, digital inclusion facilitates information flow, dismantles information barriers between urban and rural areas, and enhances rural residents’ social and economic participation. This finding diverges from perspectives that regard digital inclusion merely as a technological construct. Second, digital inclusion narrows the urban-rural education gap and provides more equitable learning opportunities by enhancing shared access to educational resources, particularly through distance learning and online education. This outcome contrasts sharply with existing literature that predominantly focuses on income disparities or physical infrastructure development. Furthermore, digital inclusion addresses healthcare disparities through optimized resource allocation, particularly expanded telemedicine services, thereby promoting more equitable public service provision. The synergistic interaction of these pathways underscores the importance of digital inclusion in urban-rural integration, advancing theoretical understanding in this field.

Third, we examine the moderating role of regional heterogeneity—specifically, the differential effects across eastern, central, and western China—on the relationship between digital inclusion and urban-rural integration. Existing literature has largely overlooked this dimension, often treating digital inclusion as a universally applicable policy instrument without accounting for contextual differences. Our analysis reveals significant heterogeneity in the effects of digital inclusion on urban-rural integration across regions, attributable to disparities in economic development levels, policy implementation effectiveness, and infrastructure availability. Specifically, the relationship between digital inclusion and the urban-rural integration index is statistically significant at conventional levels in eastern and central regions, whereas its impact remains attenuated in western areas—likely constrained by the limitations of infrastructure and policy enforcement capacity. Our findings highlight the need for region-specific digital inclusion policies aligned with local development needs to foster interregional coordination and advance nationwide urban-rural integration. These findings provide theoretically grounded support for locally tailored policy-making while refining our understanding of the role of regional heterogeneity in integration dynamics.

### Research contributions

This study addresses an empirical gap in the literature on the relationship between digital inclusion and urban-rural integration in China. While existing research has explored the theoretical frameworks of digital inclusion and its role in promoting social equity and regional development, most studies focus on developed countries and other specific regions. Few studies have conducted in-depth analysis of the practical context of urban-rural integration in the context of a developing nation like China. To address this gap, we systematically analyze Chinese data from 2006 to 2019 to explore the multidimensional role of digital inclusion in advancing urban-rural integration. Specifically, we investigate the mechanisms through which digital inclusion influences urban-rural integration across four dimensions: information flow, educational resource sharing, healthcare resource allocation, and industrial convergence. We empirically validate the effectiveness of these mechanisms through rigorous regression analysis. Furthermore, we examine regional heterogeneity, highlighting variations in the implementation effectiveness of digital inclusion across regions. We pay specific attention to disparities in policy implementation and outcomes among eastern, central, and western China, thereby enriching the regional perspective in this field. Together, these contributions expand the framework for understanding digital inclusion’s role in urban-rural integration and provide an empirical basis for policy. They directly address key literature gaps, namely the insufficient consideration of regional disparities and the lack of robust empirical evidence from developing contexts.

## Supporting information

S1 Table URLs for data access. (DOC)
