# Supplementary material for: Digital inclusion and urban-rural integration: Mechanisms and regional heterogeneity in China
Source: PLoS One. 2026 Jan 20;21(1):e0340641. doi: 10.1371/journal.pone.0340641 (PMC12818669; doi:10.1371/journal.pone.0340641)
Supplement: S1 Table — (DOC) [file pone.0340641.s001.doc]

List of URLs for data access

This study is based on publicly available, aggregated macro-level statistical data. The primary sources are official publications from Chinese government agencies. Below is a list of the key data sources and repositories where these data can be accessed or obtained.

1. Core National Statistics

National Bureau of Statistics of China (NBS) <https://www.stats.gov.cn/>

Note：The paramount and official source for national and regional statistics in China. All foundational macro-economic and social indicators used in this study are derived from the annual statistical yearbooks and bulletins published by the NBS.

1. Sector-Specific Official Statistics

（1）Ministry of Education of China (MOE)：<http://www.moe.gov.cn/>

Note：The official portal for national education policies and statistics. Education-related indicators are based on the aggregated data published in its annual statistical communiqués and reports.

1. Other Sectoral Yearbooks

Note：Data for other sectors (e.g., agriculture, industry) were sourced from the corresponding annual sectoral statistical yearbooks published under the authority of the NBS and related ministries.

3. Access Pathways for Researchers

The official statistical yearbooks (National, Provincial, Sectoral) used in this analysis are published in print and digital formats. Researchers worldwide can access them through the following legitimate channels:

1. Official NBS Website: As listed above, for browsing and referencing.
2. Academic Databases: Licensed platforms such as the China National Knowledge Infrastructure (CNKI) provide access to digitized versions of these yearbooks. Access typically requires institutional subscription.
3. Commercial Purchase:Physical copies or official e-versions can be purchased through major book retailers.(e.g., the links to JD.com ).

| 2006 China Education Yearbook Purchase Page(JD.com):<https://item.jd.com/10096521283322.html>( Link to purchase the 2006 Education Statistical Yearbook on JD.com) |
| --- |
| 2007 China Education Yearbook Purchase Page(JD.com):  <https://item.jd.com/10124105692728.html> ( Link to purchase the 2007 Education Statistical Yearbook on JD.com) |
| 2008 China Education Yearbook Purchase Page(JD.com)[:](https://item.jd.com/10155899892724.html)  <https://item.jd.com/10155899892724.html> ( Link to purchase the 2008 Education Statistical Yearbook on JD.com) |
| 2009 China Education Yearbook Purchase Page(JD.com)[:](https://item.jd.com/1107775771.html)  <https://item.jd.com/1107775771.html> ( Link to purchase the 2009 Education Statistical Yearbook on JD.com) |
| 2010 China Education Yearbook Purchase Page (JD.com)[:](../S1%20Supporting%20Information.%20URLs%20for%20data%20access.doc)  [https://3.cn/2lB-vb5A?jkl=@GDs0VAaRxf@](../S1%20Supporting%20Information.%20URLs%20for%20data%20access.doc) ( Link to purchase the 2010 Education Statistical Yearbook on JD.com) |
| 2011 China Education Yearbook Purchase Page(JD.com)[:](https://item.jd.com/10155289044448.html)  <https://item.jd.com/10155289044448.html> ( Link to purchase the 2011 Education Statistical Yearbook on JD.com) |
| 2012 China Education Yearbook Purchase Page(JD.com):  <https://item.jd.com/10119052670608.html> (Link to purchase the 2012 Education Statistical Yearbook on JD.com) |
| 2013 China Education Yearbook Purchase Page(JD.com)[:](https://item.jd.com/10119086935017.html)  <https://item.jd.com/10119086935017.html> (Link to purchase the 2013 Education Statistical Yearbook on JD.com) |
| 2014 China Education Yearbook Purchase Page(JD.com):  <https://item.jd.com/10160591649796.html>(Link to purchase the 2014 Education Statistical Yearbook on JD.com) |
| 2015 China Education Yearbook Purchase Page(JD.com)[:](../S1%20Supporting%20Information.%20URLs%20for%20data%20access.doc)  [https://item.jd.com/10163647064772.html](../S1%20Supporting%20Information.%20URLs%20for%20data%20access.doc) (Link to purchase the 2015 Education Statistical Yearbook on JD.com) |
| 2016 China Education Yearbook Purchase Page(JD.com)[:](https://item.jd.com/10147313346912.html)  <https://item.jd.com/10147313346912.html> (Link to purchase the 2016 Education Statistical Yearbook on JD.com) |
| 2017 China Education Yearbook Purchase Page(JD.com)[:](../S1%20Supporting%20Information.%20URLs%20for%20data%20access.doc)  [https://item.jd.com/14414203.html](../S1%20Supporting%20Information.%20URLs%20for%20data%20access.doc) (Link to purchase the 2017 Education Statistical Yearbook on JD.com) |
| 2018 China Education Yearbook Purchase Page(JD.com):  [https://item.jd.com/12617336.html (](../S1%20Supporting%20Information.%20URLs%20for%20data%20access.doc)Link to purchase the 2018 Education Statistical Yearbook on JD.com) |
| 2019 China Education Yearbook Purchase Page(JD.com)[:](https://item.jd.com/10119502207661.html)  <https://item.jd.com/10119502207661.html> (Link to purchase the 2019 Education Statistical Yearbook on JD.com) |

4.Note on Data Privacy:

This research utilizes exclusively aggregated, county-level or higher administrative-level statistics published by official sources. It does not involve, nor do the source publications contain, any personal identifying information of individual residents or survey participants.
